# Effects of communication skill training (CST) based on SPIKES for insurance-covered pharmacy pharmacists to interact with simulated cancer patients

**DOI:** 10.1186/s40780-017-0080-0

**Published:** 2017-04-08

**Authors:** Manako Hanya, Yoshitake Kanno, Junko Akasaki, Keiko Abe, Kazuhiko Fujisaki, Hiroyuki Kamei

**Affiliations:** 1 Faculty of Pharmacy, Meijo University, 150 Yagotoyama, Tempaku-ku, Nagoya, 468-8503 Japan; 2 Akasaki Hospital, Ibusuki, Japan; 3 Nagoya University Hospital, Nagoya, Japan; 4 Gifu University, School of Medicine, Gifu, Japan

**Keywords:** Communication skill training, SPIKES, Cancer patients, Insurance-covered pharmacy pharmacists, Roter Interaction Analysis System

## Abstract

**Background:**

With the development of pharmacotherapy and radiotherapy, cancer treatment is being shifted from surgical to outpatient services, consequently increasing insurance-covered pharmacies’ frequency of dealing with cancer patients. As the psychology of these patients is complex, it is necessary for pharmacists to educate them in consideration of their cognitive/medical and psychosocial aspects. This study analyzed cancer patient management by pharmacists working in such pharmacies and their communication skills before and after communication skill training based on SPIKES, a six-step protocol for delivering bad news, to confirm the usefulness of such training.

**Methods:**

The study involved 20 pharmacists working in insurance-covered pharmacies within Aichi Prefecture. Before and after communication skill training, role-play sessions were held using standardized patients, whose levels of satisfaction were subsequently measured. Patient management by the pharmacists was analyzed using the Roter Interaction Analysis System as a method to analyze dialogues.

**Results:**

The rate of each category, representing the pharmacists’ conversation styles when dealing with the patients, changed after communication skill training as follows: [Giving information]: decreased from 37.0 to 27.6%; [Empathy statements]: increased from 12.0 to 17.2%; and [Data gathering]: increased from 18.0 to 23.3%. The increase was particularly marked in: [Acceptance], accepting patients’ emotions and events in line with [Empathy statements]; [Promoting dialogues] as a sub-category of [Building a relationship]; and [Checks for understanding] as a sub-category of [Data gathering]. Furthermore, the results of pharmacist assessment by the patients, including their levels of overall satisfaction, showed significant correlations with [Empathy statements] and [Building a relationship].

**Conclusions:**

Communication skill training may be effective to improve pharmacists’ conversation styles to listen to patients more attentively, accept their emotions, and provide education in accordance with their needs, rather than unilaterally providing information.

**Trial registration:**

The study was approved by the Ethical Review Board of Meijo University as a research activity involving humans (approval number: H26-1).

## Background

In Japan, cancer has been the leading cause of death since 1981. This major disease developed in 1 in every 2 people, and led to death in 1 in every 3 people in 2015 [1]. The Ministry of Health, Labour, and Welfare established the Basic Plan to Promote Cancer Control Programs and Acceleration Plan for Cancer Control in 2007 and 2015, respectively, for the prevention and early identification of cancer, with the aim of reducing the mortality rate associated with it [2]. With the development of radiotherapy, oral anti-cancer agents, and molecular-targeted drugs, outpatient cancer treatment without hospitalization is currently being generalized. In Japan, medical and pharmacy services are increasingly being provided separately, and it is possible for patients to receive anti-cancer medications at insurance-covered pharmacies based on prescriptions issued by doctors. Under these circumstances, insurance-covered pharmacies’ frequency of dealing with cancer patients is also increasing, requiring pharmacists’ active commitments in cancer care, such as providing anti-cancer medication guidance, and monitoring adverse drug reactions [3–5]. However, at present, the information offered to these pharmacies is limited to that related to prescriptions, and it tends to be difficult for them to obtain sufficient information regarding patients, covering cancer notification, the details of treatment, such as doses and drug administration/washout periods, and other issues that vary among patients. As a result, there are concerns that even when patients seek information related to treatment methods or anti-cancer drugs for themselves, pharmacists may not be able to provide sufficient explanations due to a lack of patient information [4, 6].

Cancer patients bear heavy psychosocial burdens, such as anxiety, depression, and fear. Fujimori et al. [7, 8] reported that approximately 15% of cancer patients desire emotional support from medical professionals, such as alleviating their distress, and showing empathy for emotions they express. On the other hand, in another previous study [9], cancer patients without an accurate recognition of pharmacists’ roles showed anxiety over pharmacotherapy, including its effectiveness and cost, as well as related adverse events. It was also clarified that cancer patients expect pharmacists to accurately recognize individual patients’ conditions, provide appropriate pharmacotherapy, and monitor adverse drug reactions through active approaches. In short, it is important to develop insight into such patients’ psychological burdens, and provide them with appropriate education in consideration of their thoughts and needs [7].

However, a large number of cancer patients occasionally deny their disease, and experience emotional conflicts in relation to treatment, reflecting their complex psychology. Therefore, it is necessary for pharmacists to educate them while sufficiently confirming explanatory models to manage their cognitive/medical and psychosocial aspects. In a previous study [10], the use of SPIKES by pharmacists for the management of cancer patients was proposed. This model is a six-step protocol for delivering ‘bad news’, specifying appropriate communication procedures (Table 1). The American Society of Clinical Oncology also regards such communication skills as useful for doctors to deliver bad news to patients [10, 11]. In fact, in a study by John et al. [12], SPIKES was used by medical students to become able to appropriately deliver bad news to patients, and it was shown to be effective to improve their communication skills.

**Table 1:** Summary of SPIKES comprised of six steps

S (Setting up the Interview)	Arrange for some privacy Attendance of the family
P (Assessing the patient’s Perception)	Correction of wrong perceptions Questions that can be answered freely
I (Obtaining the patient’s Invitation)	Confirmation of the information that the patient demands Confirmation of what a patient thinks he/she wants to know
K (Giving Knowledge and information to the patient)	Try to use nontechnical words Periodically check the patient’s understanding
E (Addressing the patient’s Emotions with empathic response)	Let the patient know that you understand the emotion Attitude to understand the emotion of the patient
S (Strategy and summary)	Put together information to be provided and confirm it for the last time Set an aim that is achievable in the future

The present study focused on cancer patient education, and examined whether participation in communication skill training (CST) based on SPIKES is effective for pharmacists to acquire communication skills to appropriately provide such education in individual situations.

## Methods

### Subjects

Among the pharmacists working in insurance-covered pharmacies within Aichi Prefecture, who participated in the workshop < Medical Communication with Cancer Patients > held by the Aichi Pharmaceutical Association, and to whom the study objective was explained, 21 consented, and 20 (3 males and 17 females) completed the study procedure. Table 2 outlines their attributes. The study objective and methods were explained to the subjects using written documents to obtain their signed consent after confirming their sufficient understanding and agreement. The study was approved by the Ethical Review Board of Meijo University as a research activity involving humans (approval number: H26-1).

**Table 2:** Characteristics of the participating pharmacists

	Sex	Duration of registration (years)	Duty experience in the insurance pharmacy (years)	Employment form	Attendance experience of the CST
1	Female	26.0	15.0	Part-time	No
2	Female	34.0	4.0	Manager	No
3	Male	24.3	20.4	Supervisor	Yes
4	Female	24.1	8.0	Full-time	No
5	Female	30.0	12.0	Supervisor	No
6	Female	22.1	19.9	Supervisor	No
7	Male	14.0	13.0	Full-time	No
8	Female	43.3	40.0	Supervisor	No
9	Female	32.0	20.0	Full-time	No
10	Female	30.0	5.0	Full-time	No
11	Female	27.2	25.0	Supervisor	Yes
12	Female	23.1	16.0	Supervisor	Yes
13	Female	30.1	15.0	Full-time	No
14	Female	25.0	0.5	Part-time	No
15	Female	6.7	2.3	Full-time	No
16	Female	28.3	14.0	Full-time	Yes
17	Female	31.0	4.5	Supervising	Yes
18	Male	19.2	7.1	Supervisor	No
19	Female	26.3	26.3	Full-time	No
20	Female	24.1	5.0	Part-time	No
Mean ± SD		26.4 ± 7.6	13.7 ± 9.5		Yes percentage 25.0%

### Methods

**Fig. 1:**
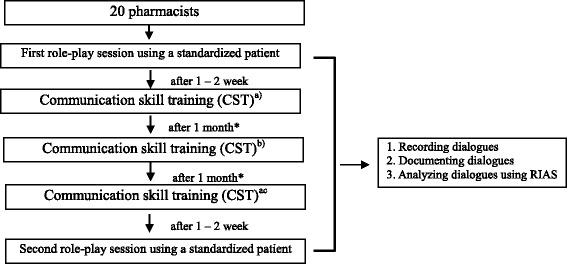
Flowchart of CST Based on SPIKES. a) Developing basic knowledge of TS-1 and communication (5 h); b) developing basic knowledge of communication with cancer patients and SPIKES using DVD, and performing role-plays with another pharmacist (5 h); and c) performing role-plays with another pharmacist and a standardized patient (5 h). *Home studies between CST sessions were not instructed

In the study, SPIKES was incorporated into CST for the management of cancer patients (Fig. 1). To confirm the usefulness of such training, a role-play session was held before and after it, using standardized patients who had been trained based a scenario to simulate cancer patients. Before the session, the role-play process was explained to the pharmacists. The duration of each session was 15 min, and the first session simulated the following situation: notifying a patient that the prescribed drugs are ready, and providing the patient, who has visited the pharmacy for the first time, with guidance on medication using the anti-cancer drug TS-1. When dealing with the patient, the pharmacists used: the relevant prescription sheet, an interview sheet for first-time patients, a handbook for patients to appropriately use the drug, and drug envelope. The second session was held within 1 month after the end of CST; although it was based on the same scenario as that used during the first session, a different standardized patient was used. The contents of both sessions were recorded using an IC recorder. Furthermore, after each session, the standardized patients assessed management by the pharmacists using an exclusive sheet (Table 3). This pharmacist assessment sheet consisted of 15 questions to be answered on a 5-point scale: from <5. Very appropriate > to <1. Very inappropriate>. In addition, the patients’ levels of overall satisfaction were measured through the question: <Overall satisfaction with management by the pharmacist to reduce the patient’s anxiety>, which was answered on a 6-point scale: from <6. Very satisfied > to <1. Very dissatisfied > .

**Table 3:** Items of pharmacist assessment by patients

Opening^a^	1	Giving considerations for the patient’s comfort, such as advising him/her to sit on a chair
	2	Considering the patient’s current physical condition, making empathy statements, and expressing appreciation for having endured examination and treatment in the hospital
Data gathering^a^	3	Asking effective (understandable) questions to more deeply understand the patient’s situation
	4	Asking questions regarding the patient’s feelings and anxiety
Patient education^a^	5	Explaining using understandable words
	6	Providing education while confirming the patient’s understanding
	7	Recognizing the patient’s anxiety accurately, and providing explanations in consideration of it
	8	Reducing the patient’s anxiety over chemotherapy
Closing^a^	9	Confirming whether or not there were other questions or issues causing anxiety
	10	Stating that consultation is available at all times
Communication^a^	11	Proceeding with the session in accordance with the patient’s pace and process
	12	Listening to the patient’s emotions related to the disease (explanatory model), complaints, and anxiety with attention (not interrupting until the end, nodding, and showing back-channel responses)
	13	Communicating with the patient non-verbally (eye contact, tone of voice, and distance)
	14	Making empathy statements in accordance with the content of the patient’s narrative
	15	Addressing the patient’s disappointment after being notified of cancer treatment as ‘bad news’ (sharing distress and thoughts, rather than consoling without much consideration)
Overall patient satisfaction^b^		Overall satisfaction with management by the pharmacist to reduce the patient’s anxiety

^a^5-point scale: from <5. Very appropriate > to <1. Very inappropriate>

^b^6-point scale: from <6. Very satisfied > to <1. Very dissatisfied>

### Analysis

The recorded dialogues between the pharmacists and patients were analyzed using the Roter Interaction Analysis System (RIAS) [13–15], a dialogue analysis method developed by Debra L. Roter, and currently being used the most widely in Western countries. In this system, dialogues are divided into utterances as the minimum unit to express thoughts and events. Subsequently, these utterances are classified into 42 categories for coding. In the present study, coding was performed by 2 coders to ensure sufficient validity, conforming to the RIAS Manual [14]. Furthermore, the RIAS categories were classified as shown in Table 4 [14]. In line with this, the category [Checks] was divided into 2 sub-categories: 1) rephrasing for confirmation of understanding, accurate communication, and clarification; and 2) confirming one’s understanding of patient’s feeling. The sub-category 2) was added to the RIAS categories as a new category, [Acceptance] (Table 4). The data were analyzed, focusing on 2 items: 1) changes in the number of the pharmacists’ utterances; and 2) the influences of increases in such a number on the patients’ levels of satisfaction. The Wilcoxon signed-rank test and single regression analysis were used to examine the former and latter, respectively, with SPSS (22.0 for Windows, SPS Co., Ltd.) for statistical processing.

**Table 4:** RIAS category in this study

Classification in this study	RIAS category
Empathy statements	
Acceptance	Confirming one’s understanding of patient’s feeling (^a^)
Emotional communication	Empathy, Shows concern or worry, Reassures, Encourages or shows optimism, Legitimizing statement
Positive talk	Laughs, tells jokes, Shows approval-direct, Gives compliment-general
Building relationship	
Remediation	Remediation, Partnership statements
Social chitchat	Personal remarks, social conversation
Initiating conversation	Shows agreement or understanding, Back-channel responses
Data gathering	
Medical data gathering	Asks question about medical condition, therapeutic regimen
Psychosocial data gathering	Asks question about lifestyle and psychosocial information
Checks for understanding	Paraphrase. Checks for understanding, Asks for permission, Bid for repetition
Patient education	
Gives information	Gives lifestyle and psychosocial information
Counsels	Gives information about medical condition, therapeutic regimen

^a^A new category added in this study

## Results

### Changes in the number of the pharmacists’ utterances after CST

**Fig. 2:**
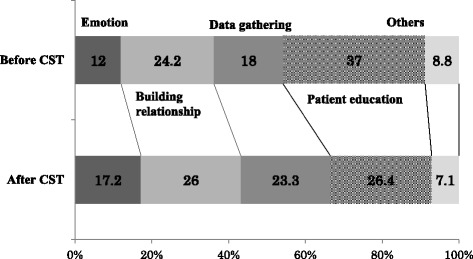
Changes in the conversation style of pharmacists after CST

Figure 2 shows the rates of each category, representing the pharmacists’ utterances before and after CST: [Giving information]: decreased from 37.0 to 27.6%; [Empathy statements]: increased from 12.0 to 17.2%; [Building a relationship]: increased from 24.2 to 26.0%; and [Data gathering]: increased from 18.0 to 23.3%. Concerning changes in the number of the pharmacists’ utterances after CST (Table 5), there were significant increases in the rates of the following categories: [Acceptance]: accepting patients’ emotions and events in line with [Empathy statements]; [Emotional talk]: showing empathy and approval, and legitimizing statements; and [Promoting dialogues]: showing agreement/understanding and back-channel responses as a sub-category of [Building a relationship]. Furthermore, the rates of [Open/closed questions: medical] and [Open/closed questions: psychosocial] as sub-categories of [Data gathering], in addition to [Checks for understanding], also markedly increased after CST. In contrast, the rate of [Counsels] as a sub-category of [Giving information] significantly decreased after it.

**Table 5:** Changes in the utterances of pharmacists after CST

RIAS category	Before CST	After CST	Difference in utterances	*P*
Empathy statements	13.8 ± 6.8	26.0 ± 10.9	12.2 ± 13.3	0.001**
Acceptance	5.4 ± 4.6	14.9 ± 8.9	9.5 ± 11.1	0.002*
Emotional communication	5.0 ± 3.8	6.7 ± 3.8	1.8 ± 3.3	0.041*
Positive talk	3.4 ± 2.5	4.4 ± 3.3	1.0 ± 3.4	0.243
Building relationship	27.9 ± 10.9	39.4 ± 11.5	11.5 ± 16.4	0.007*
Remediation	1.9 ± 1.7	3.0 ± 2.8	1.1 ± 3.4	0.207
Social chitchat	4.3 ± 1.7	3.6 ± 1.2	−0.7 ± 1.5	0.060
Initiating conversation	21.8 ± 10.0	32.9 ± 10.2	11.1 ± 15.3	0.005*
Data gathering	20.7 ± 8.1	35.3 ± 11.7	14.6 ± 10.7	0.000**
Medical data gathering	10.2 ± 5.0	17.7 ± 6.3	3.1 ± 3.3	0.003*
Psychosocial data gathering	3.3 ± 3.1	6.3 ± 3.1	7.6 ± 6.7	0.000**
Checks for understanding	1.4 ± 1.4	11.3 ± 6.0	9.9 ± 5.8	0.000**
Patient education	42.6 ± 15.2	40.0 ± 13.4	−2.6 ± 17.0	0.546
Gives information	23.2 ± 9.9	24.7 ± 8.9	1.6 ± 11.8	0.463
Counsels	19.4 ± 7.6	15.3 ± 6.1	−4.1 ± 8.1	0.013*
Others	10.1 ± 3.5	10.7 ± 2.8	0.6 ± 3.7	0.455

Wilcoxon signed rank test *N* = 20 *P < 0.05 **P < 0.001

### Relationships between pharmacist assessment by the patients and the pharmacists’ utterances (Table 6)

**Table 6:** Correlations between the number of pharmacists’ utterances and assessment of pharmacists by the patients/levels of overall satisfaction of the patients

	Assessment by patients		Overall satisfaction	
RIAS category	Correlation coefficient	*P*	Correlation coefficient	*P*
Empathy statements	0.325	0.162	0.472	0.036*
Acceptance	0.191	0.420	0.314	0.177
Emotional communication	0.408	0.074	0.467	0.038*
Positive talk	0.254	0.280	0.366	0.112
Building relationship	0.604	0.005*	0.520	0.019*
Remediation	−0.081	0.735	−0.198	0.403
Social chitchat	−0.077	0.749	−0.096	0.686
Initiating conversation	0.675	0.001**	0.613	0.004*
Data gathering	0.117	0.622	0.326	0.160
Medical data gathering	0.109	0.646	0.262	0.264
Psychosocial data gathering	0.012	0.960	0.062	0.796
Checks for understanding	0.030	0.901	0.194	0.413
Patient education	0.397	0.083	0.282	0.229
Gives information	0.318	0.172	0.292	0.212
Counsels	0.366	0.112	0.164	0.491

Single regression analysis *N* = 20 *P < 0.05 **P < 0.001

On examining the relationship between the results of pharmacist assessment by the patients and the pharmacists’ utterances, assessment scores showed significant positive correlations with [Building a relationship] and [Promoting dialogues] (*r* = 0.604 and *r* = 0.675, respectively).

As for the relationship between the patients’ levels of overall satisfaction with management by the pharmacists and the number of the latter’s utterances, the levels were significantly and positively correlated with [Empathy statements] and its sub-category [Emotional talk] (*r* = 0.472 and *r* = 0.467, respectively). Such a correlation was also observed with [Building a relationship] and its sub-category [Promoting dialogues] (*r* = 0.520 and *r* = 0.613, respectively).

## Discussion

The present study examined the effects of CST based on SPIKES for pharmacists to educate cancer patients visiting insurance-covered pharmacies in consideration of their situations. Concerning the pharmacists’ conversation styles, the rate of [Giving information] was the highest, at 37.0%, before CST, revealing their tendency to attach importance to this area when providing patient education. Currently, pharmacists’ skills to communicate with patients are being increasingly focused on, leading to the adoption of measures, such as establishing the family pharmacist system [16]. Their approaches for cancer patients tend to be limited to unilateral knowledge provision related to dosage and drug efficacy in general, as they specialize in medications, and are charged with the task of conveying information [17, 18]. In fact, before CST, the pharmacists mainly provided patient education by [Giving information], supporting the results of previous studies. However, their conversation styles changed after it, with a decrease in the rate of [Giving information] to 26.4% and increase in the number of their utterances related to [Empathy statements], [Building a relationship], and [Data gathering]. The training program used in the study integrated CST and SPIKES to facilitate information provision in consideration of patients’ needs and desires after collecting both medical and psychosocial data from them. It should particularly be noted that the steps < P(Assessing the patient’s Perception) > and < I(Obtaining the patient’s Invitation) > aim to confirm individual patients’ recognition of their disease and the contents of information they demand. Thus, in these steps, the relevant explanatory model to clarify their levels of understanding of their disease, as well as the levels of explanation they desire, is confirmed. The step < E(Addressing the patient’s Emotions with empathic response) > aims to accept patients by listening to them with empathy. In this step, medical professionals closely follow-up patients with anxiety, and address their emotions to reduce their sense of isolation and mental stress. In the present study, a DVD was created for CST based on SPIKES, and the method to effectively use this model was clearly outlined through role-play with standardized patients. The pharmacists who participated in CST watched the DVD, and performed role-plays with another pharmacist first and then a standardized patient. Furthermore, they observed patient management by other pharmacists. Through such training, they retrospectively examined the management they had performed daily, and this may have improved the quality of their approaches to address the psychology of cancer patients with empathy.

On the other hand, the patients’ levels of overall satisfaction were significantly correlated with [Empathy statements] and ‘listening to patients and showing agreement’ as part of [Building a relationship]. In studies conducted by Fujimori et al. to examine cancer patients’ preferences [7, 8], it was shown that patients desire medical professionals to emotionally support them, ensure sufficient time to ask questions, and provide simple explanations without using technical terms. Similarly, in the present study, the patients tended to be satisfied with the pharmacists’ approaches to address their emotions, including anxiety, show empathy, and establish a favorable relationship by listening to them with back-channel responses. Cancer patients begin to show psychological responses to the disease, such as anxiety and fear, when its presence is suspected. They are subject to marked mental and physical stress when they undergo unfamiliar examination and diagnosis in a hospital while being suspected of having cancer. They also develop senses of anxiety and fear due to cancer from various perspectives, including: the effects of future treatment, adverse reactions to anti-cancer drugs, progression of the disease, and influences on their living environments and other family members. As the details of such anxiety and fear vary among patients, it is necessary to provide psychological support for them in consideration of each situation. However, there is a tendency for medical professionals to generalize patients, disregard their living conditions and desires that vary among them, and attach importance to evidence-based information provision. Such a difference in thoughts between patients and medical professionals frequently causes conflicts in medical settings. White M. et al. [19] established a narrative approach, through which the latter listen to the former’s statements, recognize their thought process to clarify their desires, and provide appropriate explanations in each case. This approach aims to externalize cancer-related issues, such as the influences of the disease and its impact on families and others, and clarify individual patients’ problems, rather than focusing on their or their families’ insufficient skills or the former’s sense of being responsible for troubling other family members due to the disease. Dialogues for such externalization do not simply address patients’ emotions, but aim to identify their problems by accurately recognizing what they regard as problems and what they place value on. In SPIKES used in the present study, it is also necessary for medical professionals to confirm the appropriate explanatory model for each patient, and collect information while recognizing the fact that patients’ statements contain extensive information in the steps < P: assessing the patient’s Perception > and < I: obtaining the patient’s Invitation>. The clarification of individual patients’ thoughts and the contents of information they demand from medical professionals helps the former realize that the latter understand and listen to them, consequently contributing to trust-based relationships between the 2 parties. By establishing such relationships, it may be possible to create environments in which patients can easily consult medical professionals and actively express themselves. The pharmacists who participated in CST repeatedly learned and acquired communication skills based on SPIKES. This may have led to significant increases in the number of their utterances as empathy statements expected by patients, as well as relationship-building attitudes, indicating that they successfully created a favorable environment for patients to express their desires. The CST program developed in the study may contribute to the shift of patient education from information provision to the promotion of patients’ own statements and the establishment of favorable patient-medical professional relationships.

As a study limitation, the influences of pharmacists’ individual utterances on patients remained unclear, as the use of RIAS for analysis is limited to quantitative studies. However, the present study examined the usefulness of CST based on SPIKES to improve pharmacists’ communication skills, and confirmed it by examining the correlation between changes in the structure of their utterances and consequent patient satisfaction. In addition, as the study did not involve real cancer patients, but standardized patients who had been trained based on a scenario to simulate them, it may also be necessary to confirm whether pharmacists similarly manage the former as another future challenge. As future perspectives, it may be necessary to distribute the educational DVD used for CST based on SPIKES to insurance-covered pharmacies, and examine the effects of patient education using this model for patient management in an actual clinical setting, in addition to changes in the results when involving an increased number of samples.

## Conclusions

On examining categories representing insurance-covered pharmacy pharmacists’ styles of conversation with cancer patients, the rate of [Giving information] was the highest, at 37.0%, revealing their tendency to attach importance to this category when providing medication guidance. After CST based on SPIKES, such styles changed, and the pharmacists began to provide medication guidance while attaching importance to information gathering and emotional statements, in addition to information provision. As this also increased patient satisfaction levels, CST may be a useful method for pharmacists to become able to provide medication guidance needed by cancer patients in consideration of their conditions.
